# RTA-like proteins regulate azole susceptibility by affecting the expression of the oxidoreductase gene *oxrA* in *Aspergillus fumigatus*

**DOI:** 10.1186/s12866-026-04985-x

**Published:** 2026-04-13

**Authors:** Jing Wu, Di Su, Zhongyuan Niu, Sitong Liu, Chenxi Li, Jing Ye, Xiaogang Zhou

**Affiliations:** Anhui Key Laboratory of Infection and Immunity, Department of Microbiology, School of Basic Medicine, Bengbu Medical University, Bengbu, China

**Keywords:** *Aspergillus fumigatus*, RTA-like protein, Azole resistance, Oxidoreductase, Drug efflux pump

## Abstract

*Aspergillus fumigatus*, a saprophytic fungus and the principal etiologic agent of aspergillosis, is increasingly implicated in drug-resistant infections, posing challenges to clinical management. RTA-like proteins, a distinctive class of transmembrane fungal proteins, are involved in several stress responses. Nonetheless, their role in fungal azole resistance is yet to be determined. Transcriptomic studies performed in this investigation revealed that azole stimulation significantly upregulates the expression of RTA-like proteins in *A. fumigatus*. Phenotypic analyses indicated that relative to the wild-type strain, the *Δrta1*, *Δrta5* and *Δrta7* mutants exhibited increased sensitivity to antifungal agents, including azoles and amphotericin B (AmB). RNA sequencing and real-time quantitative polymerase chain reaction indicated a significant upregulation of the oxidoreductase gene *oxrA*, which is integral to the oxidative stress response, in the *Δrta1*, *Δrta5* and *Δrta7* mutants. Furthermore, the *oxrA* overexpression strain (OE::*oxrA*) displayed increased tolerance to oxidative stress, yet paradoxically, it also showed a considerable hike in sensitivity to azole and AmB. This heightened drug sensitivity was correlated with the downregulation of most drug efflux pump genes. In conclusion, the RTA-like proteins Rta1, Rta5 and Rta7 possibly modulate azole susceptibility in *A. fumigatus* by regulating *oxrA* expression, subsequently affecting the transcription of drug efflux pump genes. As proteins specific to fungi, members of the RTA family are promising targets for the development of novel antifungal therapeutics.

**Supplementary Information:**

The online version contains supplementary material available at 10.1186/s12866-026-04985-x.

## Introduction

Invasive pulmonary aspergillosis (IPA) is an acute fungal infection of the lungs associated with high morbidity and mortality rates in immunosuppressed individuals [1, 2]. The primary etiologic agent is *Aspergillus fumigatus*, a filamentous saprotrophic fungus prevalent in the environment [2, 3]. Beyond its role in life-threatening invasive infections that predominantly affect the lungs, *A. fumigatus* is implicated in chronic and allergic pulmonary diseases impacting approximately 8 million individuals globally [4]. Owing to its significant effects on human health, the World Health Organization listed *A. fumigatus* as one of the most critical fungal pathogens in 2022 [5].

Currently, only a limited number of drug classes that act on the cell membrane or cell wall are used for clinical antifungal therapy [6]. Azoles, polyenes, acrylamides, and echinocandins are among these drugs [7–10]. Owing to their high efficacy and low side effects, azoles have become the first-line treatment for clinical aspergillosis [11]. Azoles act by inhibiting 14α-sterol demethylase, depleting cellular sterols [12]. In many fungal species, this inhibition causes the accumulation of toxic sterol intermediates that subsequently disrupt cell membrane integrity [13]. Nevertheless, their widespread use in clinical settings, coupled with inappropriate use in industrial agriculture, has raised key concerns [14, 15]. The recent global escalation in azole resistance among clinical *A. fumigatus* isolates has substantially compromised the effectiveness of azole-based therapies for aspergillosis [16].

The mechanisms underlying azole resistance in *A. fumigatus* can be primarily classified into three categories. The first one is the mutation or overexpression of the drug target enzymes. Mutations in the *cyp51* gene have been identified in several drug-resistant strains isolated from clinical samples. These mutations modify the binding affinity between azoles and the Cyp51 protein, thereby inducing drug resistance [17, 18]. The second one is the increase in activity and upregulation of drug efflux pumps. Activation of these efflux pumps decreases intracellular drug concentrations, resulting in enhanced tolerance of *A. fumigatus* to azoles [19, 20]. The third one is the activation of cellular stress response pathways, such as those mediated by sterol regulatory element–binding protein (SrbA) and heat shock protein 90 (Hsp90) [21, 22]. Given the emergence of numerous drug-resistant strains of unknown origin, additional resistance mechanisms must be elucidated to provide a theoretical foundation for the clinical management of aspergillosis and the development of novel drug targets [23].

RTA-like (*R*esistance *t*o 7-*a*minocholesterol) proteins constitute a family of membrane proteins that are exclusive to fungi, distinguished by unique structural attributes and essential biological functions [24]. These proteins belong to a fungal-specific gene family that was initially identified in *Saccharomyces cerevisiae* [25]. These proteins have multiple transmembrane domains—often seven—and are highly conserved across fungal species but are absent in humans and other mammals. According to literature reports, RTA-like proteins play a role in fungal resistance against environmental stresses. For instance, exposure to amphotericin B (AmB) leads to elevated expression of RtaA, whereas the loss of RtaA leads to increased sensitivity to AmB in *A. fumigatus* [26]. Similarly, deletion of RtaA enhances susceptibility to the antifungal sterol analog 7-aminocholesterol in *S. cerevisiae* and *Candida glabrata* [27]. These findings suggest that RTA-like proteins play a broader role in fungal stress responses. However, the association between RTA-like proteins and azole tolerance in *A. fumigatus* remains largely unexplored.

In this study, RNA sequencing (RNA-Seq) analysis was performed to profile the transcriptional response of *A. fumigatus* to azoles. The findings indicated upregulation of a series of RTA-like proteins. Given that this protein family is exclusive to fungi and absent in mammals, targeting it holds promise for antifungal drug development with minimal risk of direct toxicity to human cells.

## Results

### RTA-like proteins were significantly upregulated in the *A. fumigatus* strain under azole stimulation

**Fig. 1:**
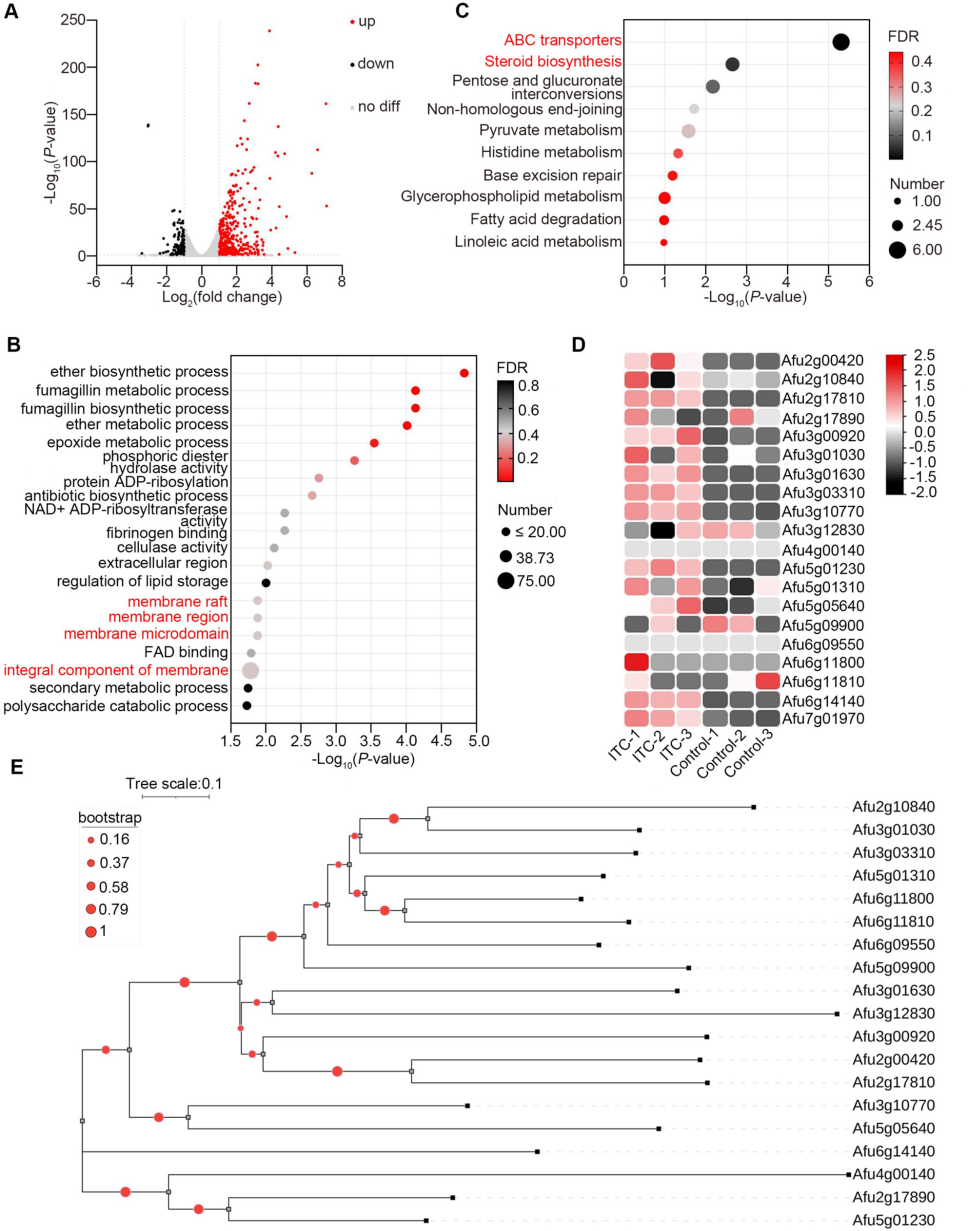
Transcriptomic analysis of the WT strains with or without azole stimulation. **A** Volcano plot of DEGs in the WT strain with 0.05 µg/mL ITC treatment compared with the WT strain without ITC treatment; **B** Gene Ontology (GO) analysis and **C** pathway enrichment analysis using the KEGG database for the DEGs; **D** Heatmap analysis of selected RTA proteins in RNA-Seq data; **E** Phylogenetic tree analysis of selected RTA homologous proteins in *A. fumigatus* using MEGA 7 software

To identify genes involved in the fungal response to azoles, RNA-Seq was performed on wild-type (WT) strains cultivated in liquid minimal medium (MM) with or without 0.05 µg/mL itraconazole (ITC). Expression levels were normalized using FPKM (fragments per kilobase per million fragments). Differentially expressed genes (DEGs) were identified using the criteria of log_2_ fold change ≥ 1 and *P*-value ≤ 0.05. As depicted in Fig. 1A, volcano analysis revealed a total of 580 DEGs, of which 122 were downregulated, and 458 were upregulated(Data set 1). The number of upregulated DEGs was notably higher than that of downregulated ones. Gene Ontologewy (GO) enrichment analysis indicated that these DEGs were involved in biological functions mainly related to cell membrane structures (marked in red), such as cell membrane components, microdomains, and membrane rafts (Fig. 1B). Kyoto Encyclopedia of Genes and Genomes (KEGG) pathway analysis demonstrated that these DEGs played a role in several metabolic signaling pathways, including ATP-binding cassette (ABC) transporters and steroid biosynthesis, both of which are critical for azole response (Fig. 1C; Data set 1). Many upregulated DEGs included RTA domain–containing proteins. These findings suggest that RTA proteins participate in azole resistance in *A. fumigatus*. Heatmap analysis confirmed significant upregulation of these RTA proteins following azole treatment (Fig. 1D). Phylogenetic analysis revealed high homology among the selected RTA proteins in *A. fumigatus* (Fig. 1E).

### RTA-like proteins are involved in multiple drug responses

To identify the most closely related RTA proteins in *A. fumigatus*, a BLASTp alignment was performed against the *A. fumigatus* genome database using *S. cerevisiae* Rta1 as the query. The search yielded seven conserved Rta homologs in *A. fumigatus* with gene access numbers of Afu5g01230, Afu2g17890, Afu6g14140, Afu3g01030, Afu1g11800, Afu6g11810, and Afu2g00420, which were named Rta1, Rta2, Rta3, Rta4, Rta5, Rta6, and Rta7, respectively. To further investigate the function of RTA proteins in *A. fumigatus*, full-length deletion mutants of these RTA genes were generated using homologous recombination. Colony phenotype analysis indicated that among the seven RTA deletion mutants, the *Δrta2* mutant exhibited a notably more fluffy colony morphology compared to the WT, whereas the other mutants did not display significant phenotypic deviations from the WT control. Furthermore, statistical analysis demonstrated no significant differences in conidial production or biomass between the RTA deletion mutants and the WT control.(Fig. S1). These findings imply that RTA proteins perform distinct functions in the growth and spore production in *A. fumigatus*.

**Fig. 2:**
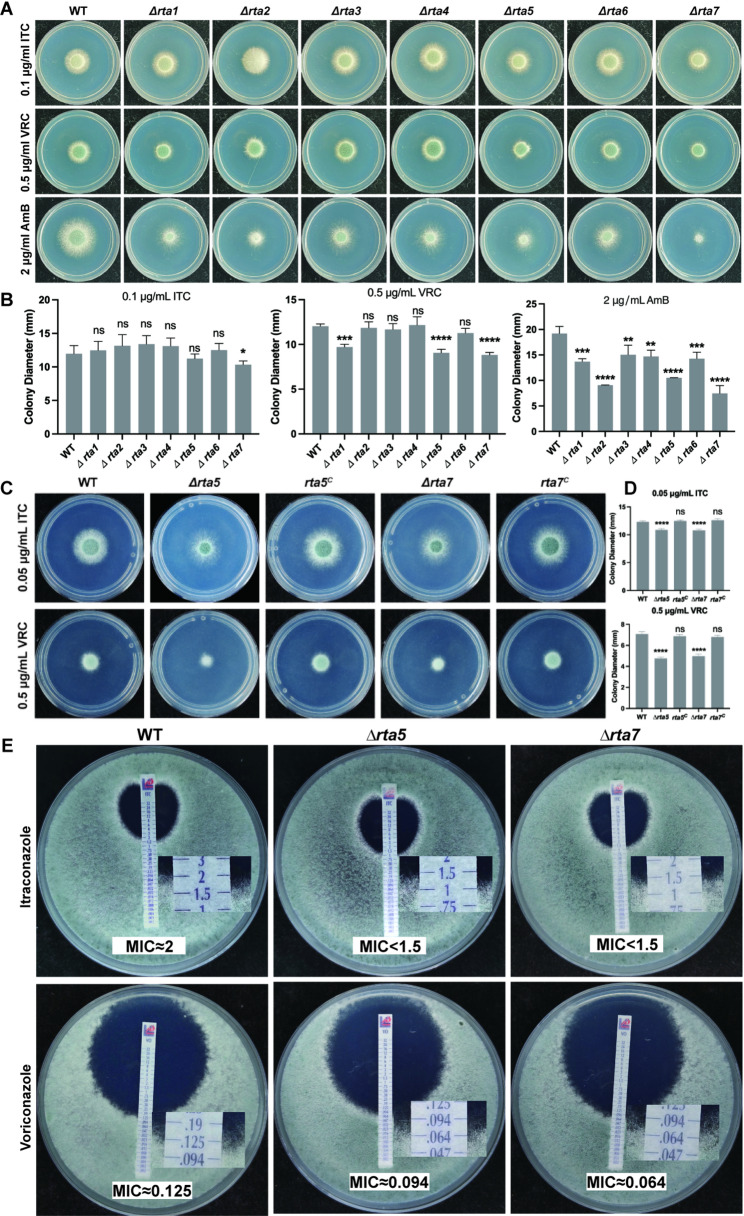
RTA-like proteins are involved in multiple drug responses. **A**,** B** Colony growth and diameter measurements of WT, *Δrta1–Δrta7* strains on media containing 0.1 µg/mL ITC for 2 days, 0.5 µg/mL VRC for 2.5 days, or 2 µg/mL AmB for 3 days. **C**,** D** Complementation analysis of the *Δrta5* and *Δrta7* strains confirms the azole susceptibility phenotype. Strains were incubated at 37 °C for 48 h. **E** The MIC values of the indicated strains were determined using commercial E-test strips. Conidia (1 × 10⁵) of WT, *Δrta5*, and *Δrta7* strains were mixed in MM medium. ITC or voriconazole E-test strips were placed on the plates, and cultures were incubated at 37 °C for 48 h.Data are mean ± SD from three independent experiments. Statistical significance was determined by one-way ANOVA with Tukey’s test .(ns, not significant; *, *P* < 0.05; **, *P* < 0.01; ***, *P* < 0.001; ****, *P* < 0.0001)

RNA-Seq data indicated that RTA-like proteins were markedly upregulated under azole stimulation. Hence, RTA-like proteins may be involved in the fungal response to azoles. To explore the role of RTA-like proteins in the antifungal drug response of *A. fumigatus*, drug susceptibility tests were conducted on the WT control and the related RTA protein deletion mutant strains. Colony phenotype and statistical analysis showed that among the seven RTA-like protein deletion mutant strains, only *Δrta7* exhibited sensitivity to ITC compared with the WT strain (Fig. 2A and B). Moreover, *Δrta1*, *Δrta5*, and *Δrta7* exhibited more growth defects than the WT strain under voriconazole (VRC) culture conditions (Fig. 2A and B). All seven deletion mutants were significantly smaller than the control strain under AmB exposure (Fig. 2A and B). This finding indicates that RTA-like proteins are involved in the antifungal response of *A. fumigatus*. To further investigate the impact of Rta5 and Rta7 on the susceptibility of *A. fumigatus* to azole drugs, we generated complementation strains of the *Δrta5* and *Δrta7* mutants. As shown in Fig. 2C and D, the *Δrta5* and *Δrta7* mutants exhibited reduced colony sizes under ITC and VRC conditions compared to the WT and complemented strain controls. Subsequently, we determined the minimum inhibitory concentration (MIC) values for the *Δrta5* and *Δrta7* mutants using commercial E-test strips. As depicted in Fig. 2E, the MIC values for ITC in the *Δrta5* (less than 1.5 µg/mL) and *Δrta7* (less than 1.5 µg/mL) mutants were significantly lower than those observed in the WT strain (approximately 2 µg/mL). Similarly, using voriconazole E-test strips, the *Δrta5* (approximately 0.094 µg/mL) and *Δrta7* (approximately 0.064 µg/mL) mutants demonstrated reduced MIC values compared to the reference WT (approximately 0.125 µg/mL). These MIC assessments further corroborate the involvement of Rta5 and Rta7 in the azole antifungal response in *A. fumigatus*.

### All seven RTA-like proteins participate in the Congo red response, whereas only Rta2 is implicated in the osmotic response in *A. fumigatus*

**Fig. 3:**
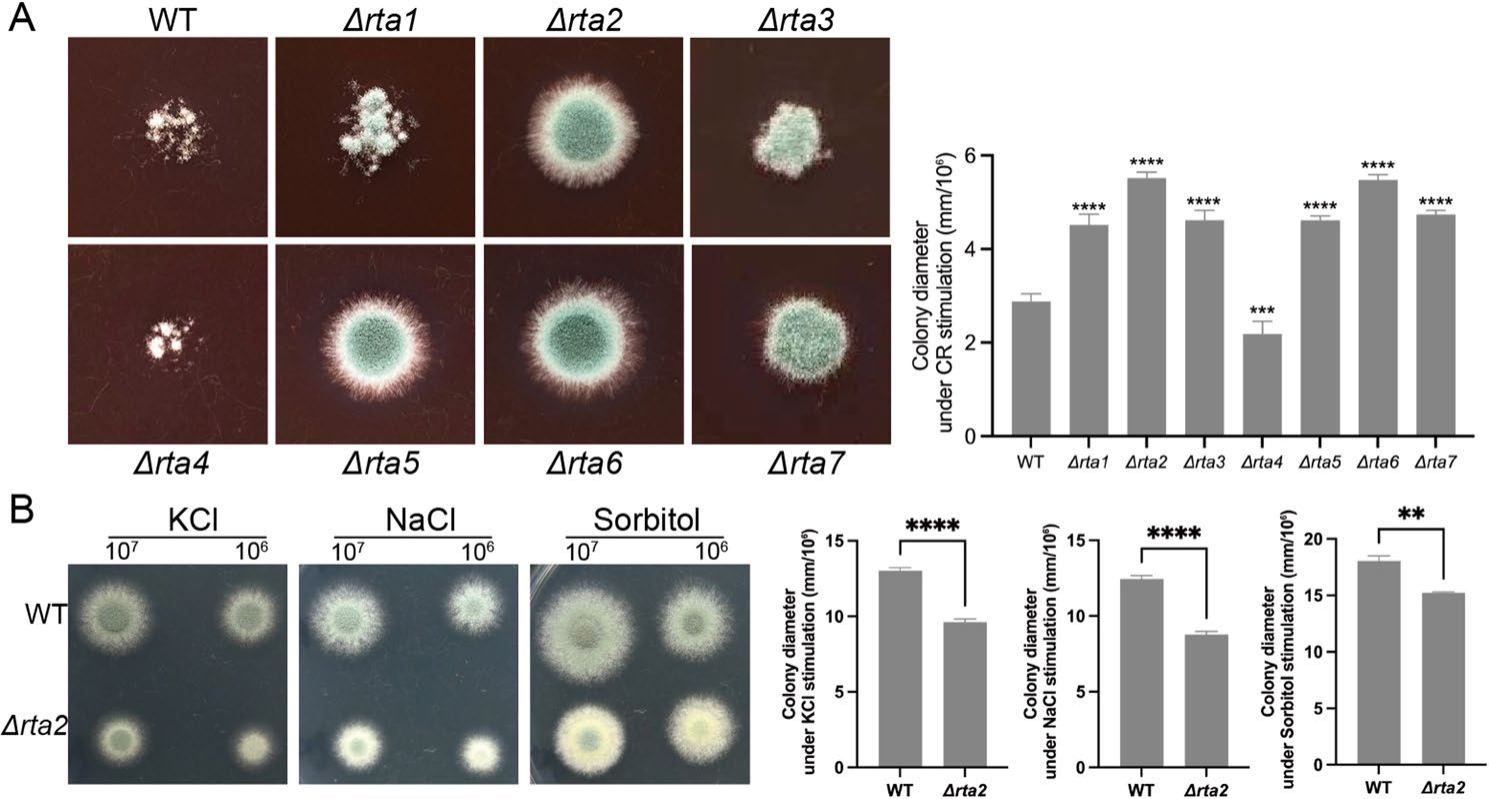
RTA-like proteins are required for the stress response of *A. fumigatus*. **A** Colony phenotype (left) and quantitative analysis (right) of WT and *Δrta* deletion strains (Δ*rta1* to Δ*rta7*) grown on MM plates supplemented with 200 µg/mL Congo red at 37 °C for 2.5 days. **B** Spot dilution assays (left) and quantitative analysis (right) of WT and Δ*rta2* strains grown on MM plates supplemented with 1 M KCl, 1 M NaCl, or 1.2 M sorbitol at 37 °C for 2 days. Data are presented as the mean ± standard deviation (SD) of three independent biological replicates. Statistical significance was determined using unpaired two-tailed Student’s t-test and one-way ANOVA with Tukey’s test.(**P < 0.01, ***P < 0.001, ****P < 0.0001.)

Osmotic stress response and cell wall integrity are the core factors closely associated with the global pathogenicity network. To further examine the function of RTA-like proteins, the WT control and the Rta deletion mutant strains were cultured in solid MM with or without osmotic stress reagents, including sorbitol, potassium chloride (KCl), and sodium chloride (NaCl), and with Congo Red (CR), a cell wall–destructive reagent (Fig. 3). The *Δrta1*, *Δrta2*, *Δrta3*, *Δrta5*, *Δrta6*, and *Δrta7* strains exhibited a CR-resistant phenotype relative to the WT strain. Conversely, the *Δrta4* strain displayed a CR-sensitive phenotype compared with the WT strain (Fig. 3A). Colony phenotype analysis revealed that of the Rta deletion mutants, only the *Δrta2* strain was more sensitive to osmotic stress (Fig. 3B). In contrast, the others showed no significant difference compared with the WT control (SFig 2). These results indicate that RTA-like proteins are necessary for the response of *A. fumigatus* to osmotic stress and cell wall stress, although their functions are not identical.

### The oxidative stress–related gene *oxrA* was overexpressed in *Δrta1*, *Δrta5* and *Δrta7* deletion mutants with increased azole sensitivity

**Fig. 4:**
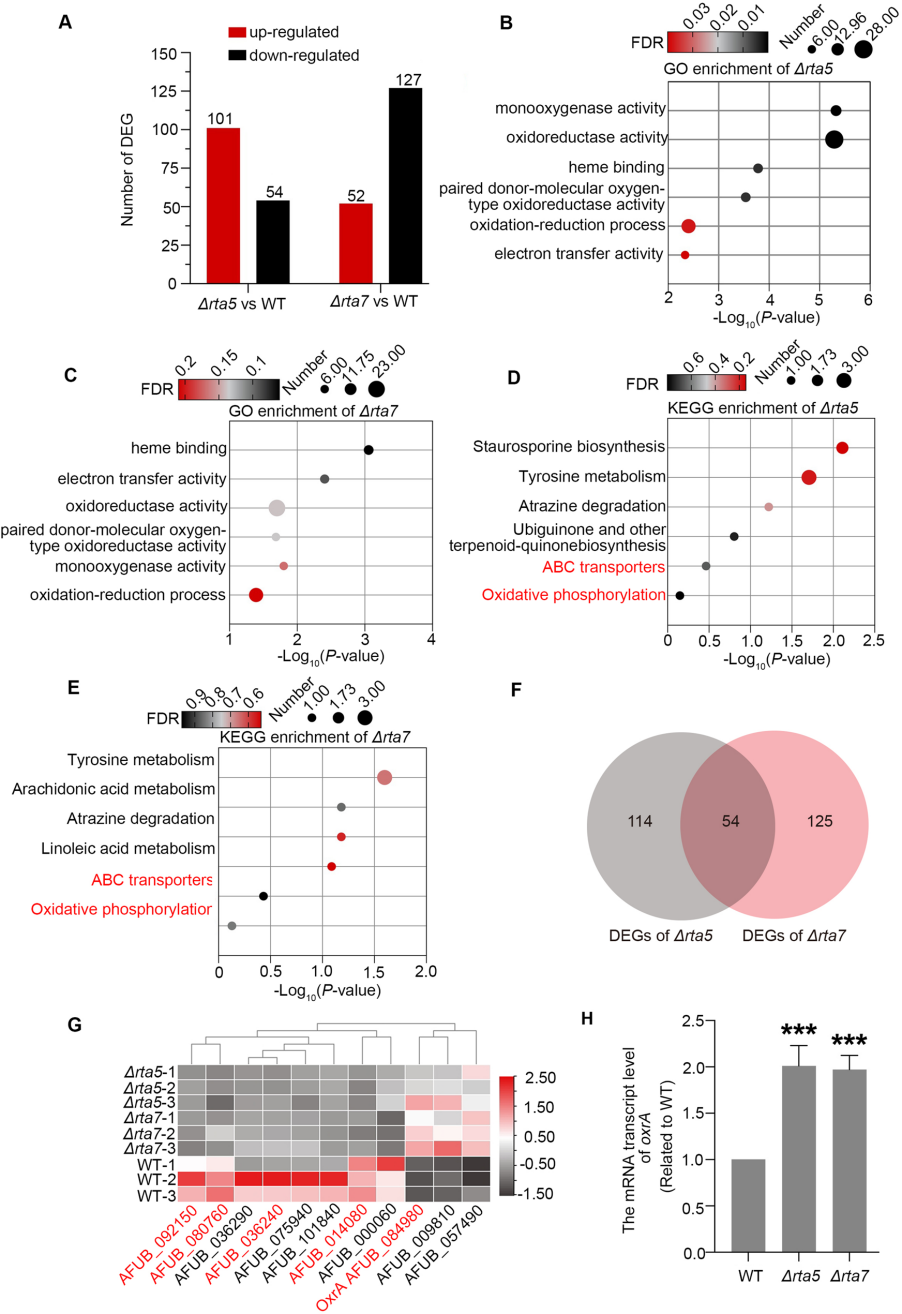
RNA sequencing analysis of the *Δrta5*, *Δrta7*, and WT strains. **A** Statistics of the DEGs in *Δrta5* and *Δrta7* strains compared with the parental WT strain; **B**,** C** Gene Ontology (GO) analysis of the DEGs functional categories enrichment for *Δrta5*(B*)* and *Δrta7 *(**C**) strains; **D**,** E** Kyoto Encyclopedia of Genes and Genomes (KEGG) analysis of pathway enrichment for *Δrta5 *(**D**) and *Δrta7 *(**E**) strains; **F** Venn diagram analysis of the differentially expressed genes (DEGs) in Δrta5 and Δrta7 strains (**G**) Heatmap comparing RNA sequencing data between the *Δrta5* mutant and the *Δrta7* mutant; **H** Real-time quantitative PCR analysis the expression levels of the *oxrA* gene in *Δrta5* and *Δrta7* strains compared with the WT strain. (***,*P* < 0.001)

Colony phenotype analysis revealed that among the deletion mutants of the RTA-like protein, the *Δrta1*, *Δrta5* and *Δrta7* mutant strains were more sensitive to azoles than the WT control (Fig. 2). To further investigate the molecular mechanism by which the RTA-like protein regulates fungal azole susceptibility in *A. fumigatus*, we selected the *Δrta5* and *Δrta7* mutant strains, which exhibited the most pronounced increase in azole sensitivity, for transcriptome sequencing. This analysis revealed that the *Δrta5* mutant had a total of 155 DEGs compared with the WT control. Of these genes, 102 were upregulated, and 54 were downregulated, with the number of upregulated DEGs significantly higher than that of downregulated DEGs (Fig. 4A). In contrast to the transcriptional profile of the *Δrta5* strain, the *Δrta7* mutant exhibited 52 upregulated DEGs and 127 downregulated DEGs compared with the WT strain (Fig. 4A; Data set 2). The number of downregulated DEGs was substantially greater than that of upregulated DEGs. GO analysis showed that the DEGs induced by *Δrta5* (Fig. 4B) and *Δrta7* (Fig. 4C) were enriched in similar biological functions, mainly oxidoreductase activity, monooxygenase activity, heme binding, and electron transfer activity, all of which are related to oxidation–reduction processes. Pathway analysis using the KEGG database demonstrated that these DEGs were involved in multiple metabolic signaling pathways (Fig. 4D, E). The DEGs in both *Δrta5* and *Δrta7* mutants were significantly enriched in pathways related to ABC transporters and oxidative phosphorylation(Fig. 4D, E; Data set 2). Thus, *rta5* and *rta7* may regulate fungal azole susceptibility via a similar mechanism. The Venn diagram analysis revealed that *Δrta5* and *Δrta7* share a set of 54 DEGs (Fig. 4F). Subsequent heatmap analysis identified several common genes between the DEGs of *Δrta5* and *Δrta7*, including RTA-like proteins, drug efflux pumps and the oxidation–reduction related protein (red marked) (Fig. 4G), of which *oxrA* (*ox*idation *r*esistance, accession number AFUB_084980), an NAD/FAD(P)-dependent oxidoreductase, was significantly upregulated in both the *Δrta5* and *Δrta7* deletion mutants. To validate the RNA-seq results, the RT-qPCR was performed using biological replicate samples from the *Δrta5*, *Δrta7*, and the WT control. As illustrated in Fig. 4H, the expression of *oxrA* was upregulated in both the *Δrta5* and *Δrta7* mutants. To determine whether other RTA proteins involved in azole susceptibility regulation similarly influence *oxrA* expression, additional RT-qPCR experiments were conducted. As demonstrated in Supplementary Fig. 3, consistent with the findings for the *Δrta5* and *Δrta7* mutants, *oxrA* expression was also significantly upregulated in the *Δrta1* mutant strain. Combined with literature reports that the oxidation–reduction process is crucial for the antifungal response [28], *oxrA* may contribute to azole susceptibility in the *Δrta1*, *Δrta5* and *Δrta7* mutants.

### Overexpression of *oxrA* enhances azole susceptibility and downregulates the expression of the drug efflux pumps

**Fig. 5:**
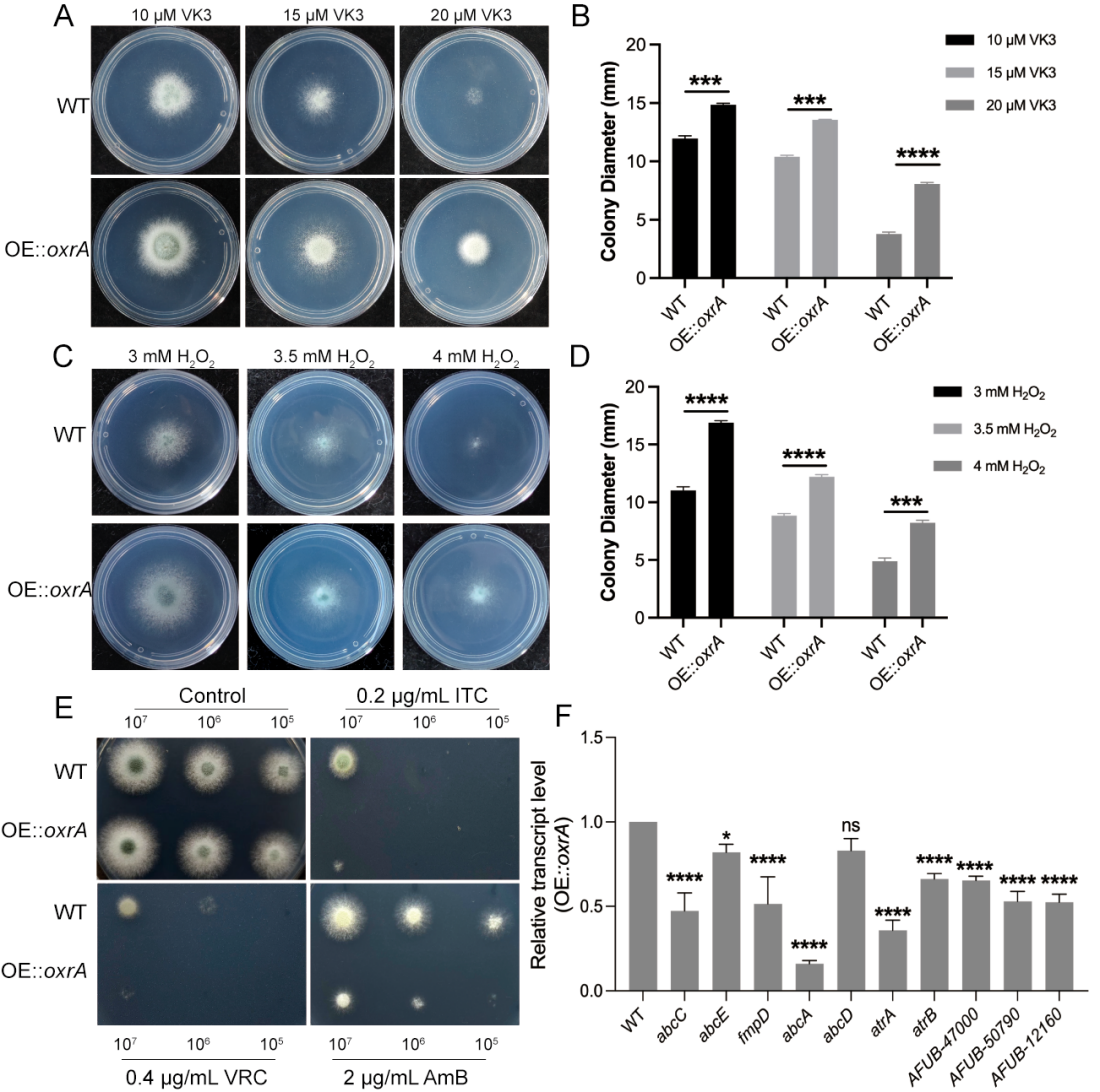
*OxrA* is required for oxidative stress and azole susceptibility in *A. fumigatus*. **A** Colony phenotype of the WT and OE::*oxrA* strains grown on MM plates supplemented with 10, 15, or 20 µM VK3 at 37 °C for 2 days. **B** Quantitative analysis of colony diameters corresponding to the results in panel (**A**). **C** Colony phenotype of the WT and OE::*oxrA* strains grown on MM plates supplemented with 3 mM, 3.5 mM or 4 mM H₂O₂ at 37 °C for 2.5 days. **D** Quantitative analysis of colony diameters corresponding to the results in panel (**B**). **E** Colony phenotype of the OE::*oxrA* and the WT control cultured on media containing antifungal drugs (0.2 µg/mL ITC, 0.4 µg/mL VRC, and 2 µg/mL AmB) at 37 °C for 2 days; **F** Real-time quantitative PCR comparison of indicated genes between the OE::*oxrA* strain and its parental WT strain. Data are presented as the mean ± standard deviation (SD) of three independent biological replicates. Statistical significance was determined using an unpaired two-tailed Student’s t-test.( ns, not significant ; *,*P* < 0.05; ***,*P* < 0.001; ****,*P* < 0.0001)

To investigate the functional conservation of *A. fumigatus oxrA*, an attempt was made to construct a full-length deletion mutant strain. However, this attempt was unsuccessful. Hence, the overexpression strain of *oxrA* (OE::*oxrA*) was constructed. Research has shown that *oxr1*, the *oxrA* homolog in humans, confers protection against oxidative stress and plays a role in the regulation of mitochondrial function [29]. To verify the role of *oxrA* in fungal response to oxidative stress, the OE::*oxrA* and the WT control were cultured on MM with or without the oxidative stress agents hydrogen peroxide (H₂O₂) and vitamin VK3 (VK3). As illustrated in Fig. 5, colony phenotype analysis revealed that the OE::*oxrA* strain exhibited increased tolerance to the oxidative stress agents VK3 (Fig. 5A and B) and H₂O₂ (Fig. 5C and D) compared with the WT control. Therefore, *oxrA* appears to be required for the oxidative stress response of *A. fumigatus*. *OxrA* was upregulated in RNA-Seq data of the *Δrta1*, *Δrta5* and *Δrta7* mutant strains, and loss of *Δrta1*, *rta5* and *rta7* increased azole susceptibility in *A. fumigatus.* Hence, the roles of *oxrA* in fungal azole response were further investigated. In drug plate assays, treatment with ITC and VRC considerably inhibited the growth of the OE::*oxrA* strain compared with the WT strain (Fig. 5E). In addition, the overexpression of *oxrA* increased the sensitivity to the polyene antifungal drug AmB (Fig. 5E). This finding aligns with the phenotypes observed in the *Δrta1*, *Δrta5* and *Δrta7* strains, suggesting that increased susceptibility to azoles in these strains may be mediated by an effect on the oxidative stress response. Numerous reports have observed that the fungal oxidative stress response influences antifungal drug susceptibility by modulating the expression of drug efflux pumps [20, 30, 31]. To ascertain the role of *oxrA* in the regulation of drug efflux pump expression, real-time quantitative polymerase chain reaction (RT-qPCR) was conducted to determine the expression levels of selected key drug efflux pumps, including *abcA*, *abcC*, *abcD*, *abcE*, *atrA*, *atrB*, and *fmpD*, and three putative drug efflux pumps (AFUB_47000, AFUB_50790, and AFUB_12160) in OE::*oxrA* and WT strains. As depicted in Fig. 5F, except for *abcD*, the expression levels of the selected drug efflux pump genes were significantly downregulated. Thus, the upregulation of *oxrA* expression may suppress the expression of the drug efflux pumps, potentially leading to increased intracellular drug concentrations and subsequent inhibition of colony growth in *A. fumigatus*.

## Discussion

### RTA-like proteins play important roles in fungal multidrug response

Recent studies have detected 23 RTA-like proteins in the pathogenic filamentous fungus *A. fumigatus* [32]. In this study, transcriptome sequencing revealed that RTA-like proteins are broadly upregulated in response to azole stimulation (Fig. 1). Phenotypic analyses of colonies indicated that compared with the WT control, three of the seven RTA deletion mutant strains (*Δrta1*, *Δrta5* and *Δrta7*) demonstrated increased sensitivity to azoles with limited phenotypic penetrance among RTA-like proteins mutants (Fig. 2). Furthermore, recent findings have shown that two other RTA family proteins—RtmA and RtmB—are also upregulated under azole exposure and that their deletion enhances azole sensitivity [32]. These findings suggest that a class of RTA-like proteins play a crucial role in fungal response to azoles. Notably, in addition to azole drugs, all RTA-like protein deletion mutants in this study showed increased sensitivity to the polyene drug AmB (Fig. 2A and B). In other *Aspergillus* species, such as *A. terreus* and *A. lentulus*, RtaA expression also increases upon AmB stimulation [26]. Previous studies have observed that the deletion of RtaA increases the sensitivity of *A. fumigatus* to AmB and AmBisome [26], whereas its overexpression improves tolerance, although it does not influence tolerance to azoles. Those findings suggested that RTA-like proteins may have broader functions in polyene resistance that are not fully explored. Furthermore, in *S. cerevisiae*, *C. glabrata*, and *Cryptococcus neoformans*, the loss of RtaA leads to heightened sensitivity to the antifungal sterol 7-aminocholesterol [24, 25, 33]. In budding yeast, the expression of Rta1 is linked to increased cellular tolerance to myriocin [33]. These results collectively suggest that RTA-like proteins in fungi are crucial for multidrug resistance, with different RTA-like proteins serving specific functions against various antifungal agents. RTA proteins with similar functions may complement each other in terms of functionality.

### RTA-like proteins are crucial for sphingolipid synthesis, yet they do not influence Cyp51 expression

Previous studies have also stated that RTA proteins in various species contribute to resistance against 7-aminocholesterol, an ergosterol synthesis inhibitor [24]. And the azole drugs exert their fungicidal effects primarily by inhibiting the activity of the ergosterol synthesis enzyme Cyp51. Thus, we hypothesize that RTA proteins regulate *A. fumigatus* azole drug susceptibility by modulating the expression of the azole target cyp51. However, transcriptomic data indicated that Cyp51 levels did not change in *Δrta5* and *Δrta7* mutants (Fig. S4). Previous studies reported that the presence or absence of RTA1 did not influence sterol accumulation [25, 34], implying that RTA proteins may affect azole tolerance via mechanisms other than altering drug target expression.

Recent studies have explored the role of sphingolipids in fungal azole tolerance and pathogenicity [35, 36]. In yeast, Rta1 is part of the lipid-translocating exporter family, aiding in exporting toxic lipophilic compounds [33, 37]. Rta1 overexpression affects responses to phytosphingosine, a sphingolipid precursor, and overall sphingolipid metabolism [38]. The homolog Rsb1 acts as a transporter or floppase for sphingolipid components, especially long-chain bases [33, 39]. Deleting Rsb1 decreases yeast tolerance to phytosphingosine. Moreover, deleting *rta1* in budding yeast increases sensitivity to myriocin, an inhibitor of sphingolipid synthesis [33]. In *C. neoformans*, Rta1 influences stress responses by modulating cell membrane function via lipid transport, whereas in *Cryptococcus deuterogattii*, it is involved in vesicle secretion [24, 40, 41]. Additionally, resent study showed that Rta3, a member of the Rta1-like family of lipid-translocating exporters specifically regulates phosphatidylcholine asymmetry and biofilm formation in *C. albicans* [42]. Collectively, these findings suggest that RTA-like proteins are vital for sphingolipid synthesis and transport in fungi. This function may also partly explain how Rta1 affects azole drug sensitivity.

### RTA-like proteins regulate azole susceptibility by affecting the fungal oxidative stress response

Oxidative stress response plays a key role in various cellular processes in fungi, including signal transduction, host interaction, and drug tolerance [43–45]. The transcriptomic analysis performed in this study and RT-qPCR validation revealed that the oxidoreductase *oxrA* was upregulated in the *Δrta1*, *Δrta5* and *Δrta7* mutants (Fig. 4G and H and SFig 3). To rigorously assess whether the regulation of azole resistance by the Rta-like protein is mediated via the upregulation of OxrA, we systematically engineered double-deficient strains lacking both *rta* gene and *oxrA*. Regrettably, these attempts were unsuccessful. Given the critical role of oxidoreductase, it is plausible to hypothesize that *oxrA* is an essential gene. Consequently, an *oxrA* overexpression strain was developed for further investigation. OxrA overexpression conferred resistance to oxidative stressors such as H_2_O_2_ and VK3, but it also heightened the sensitivity to azole drugs (Fig. 5). A recent study has demonstrated that an additional oxidoreductase, designated as AFUA_7G00700, is implicated in gliotoxin self-protection. Mutants with deletions of this gene exhibited resistance to gliotoxin, suggesting that the expression of oxidoreductase is critical for fungal stress resistance [46]. The oxidative stress response is closely related to mitochondrial function [47]. Resent studies showed that mitochondrial dysfunction can paradoxically increase azole resistance through efflux pump upregulation [48]. Prior research has indicated that oxidative stress can activate the mitochondrial-related transcription factor Pdr1, which then promotes the expression of pleiotropic drug resistance (PDR) genes, including drug efflux pumps, influencing fungal drug resistance [49–51]. In yeast, RTA1 is known as a downstream target of Pdr1 that controls antifungal drug tolerance [38]. The RT-qPCR data also showed that OxrA overexpression caused a downregulation of many drug efflux pump genes (Fig. 5F). This finding implies that the RTA-like proteins may regulate the resistance to azole drugs through the RTA-oxidative stress response (mitochondrial dysfunction)- drug efflux pump geometric network in *A. fumigatus*. Overall, this result highlights the significant conservation of RTA-like proteins across fungi and their absence in mammals, which makes them promising targets for antifungal therapies [26, 37]. These proteins are highly conserved, contain multiple transmembrane domains, and participate in various drug responses without homologs in humans or other mammals, making them ideal candidate drug targets.

## Materials and methods

### Strains, media, and culture conditions

All *A. fumigatus* strains utilized in this study are listed in Table 1. Two sterile media formulations were employed for experimental cultures: MM (basal medium) and YAG medium. MM comprised 50 mL/L sterile 20× salt solution, 1% (w/v) glucose, 1 mL/L trace element solution, and 2% (w/v) agar, with the final pH adjusted to 6.5 using sterile 1 M NaOH. YAG medium contained 0.5% (w/v) yeast extract, 2% (w/v) glucose, 1 mL/L trace element solution, and 2% (w/v) agar [52]. For liquid culture experiments, all media were prepared without agar supplementation. All strains were incubated at 37 °C in a constant-temperature incubator, and the culture duration was strictly controlled to ensure consistency across experimental replicates.

**Table 1:** Strains used in this study

Strain	Genotype	Source
A1160	Δku80, pyrG	LL-lab
WT	Δku80, A1160::pyrG	LL-lab
Δrta1	Δku80, pyrG, Δrta1::pyrG	This study
Δrta2	Δku80, pyrG, Δrta2::pyrG	This study
Δrta3	Δku80, pyrG, Δrta3::pyrG	This study
Δrta4	Δku80, pyrG, Δrta4::pyrG	This study
Δrta5	Δku80, pyrG, Δrta5::pyrG	This study
Δrta6	Δku80, pyrG, Δrta6::pyrG	This study
Δrta7	Δku80, pyrG, Δrta7::pyrG	This study
rta5^C^	Δku80,pyrG,Δrta5::pyrG, rta5::rta5::hyg	This study
rta7^C^	Δku80,pyrG,Δrta7::pyrG, rta7::rta7::hyg	This study
OE::*oxrA*	∆ku80, pyrG, gpd:: *oxrA*:: pyrG	This study

### Construction of *rta* deletion mutants

All oligonucleotide primers used in this study are detailed in Table 2. To produce *rta* deletion strains, the open reading frame (ORF) of the *rta* gene was replaced with the *pyrG* selectable marker via homologous recombination. Initially, the *pyrG* marker gene was amplified from the plasmid pXDRFP4 using the primer pair PyrG-F/PyrG-R. Separately, approximately 1.5 kb of upstream and downstream flanking sequences of the *rta* ORF were amplified using gene-specific primer pairs: the upstream flanking sequence was obtained with the primers rta-P1/rta-P3, whereas the downstream flanking sequence was amplified with the primers rta-P4/rta-P6. These purified fragments were subsequently combined as templates for overlap extension PCR using the primer pair rta-P2/rta-P5 to construct the full-length *rta* deletion cassette. The resulting fusion product was used to transform the recipient strain A1160 using a standard protoplast transformation protocol [53]. The transformants were initially selected on MM (without uracil supplementation, to select for *pyrG* expression) and then verified using diagnostic PCR. For PCR verification, three primer pairs were utilized to confirm correct integration: rta-P1/PyrG-R (to validate upstream integration), rta-self-F/rta-self-R (to confirm *rta* ORF deletion), and PyrG-F/rta-P6 (to verify downstream integration). The same construction and verification protocol was used to generate all seven *rta* deletion strains.

**Table 2:** Primers used in this study

Name	Sequence(5’→3’)
rta1-P1	CGTCAAGGTGCTGATAGTC
rta1-P2	GTACCTGGCGTGATTGATATG
rta1-P3	GGTGAAGAGCATTGTTTGAGGCGCTGACCATGATGAGAATTTTCCAG
rta1-P4	CATCAGGCCTCCTCTCAGACAGCTCACGGGCCTGATATCTAAGG
rta1-P5	GGATTTGCCTTGCACCTTGAG
rta1-P6	GATCATGCCAATCGACGCATG
rta1-Up	TTCACTACAACCCATTTCCGT
rta1-Down	TTCTGTGCCTTGAGTTCCATC
rta2-P1	GACCCGGTTTGGAATGGTAACGA
rta2-P2	ACTTACCATCTGCACCCTACCT
rta2-P3	GGTGAAGAGCATTGTTTGAGGCGGCTGCTCTCCTGTTCAGAGAAG
rta2-P4	CATCAGGCCTCCTCTCAGACAGCACGACCATTCGAAGTTGGGTG
rta2-P5	CCCCATCTCCATGATCAATTCC
rta2-P6	TGGTTGATAGGTACGGTAGAAG
rta2-Up	ATGCCTACTCCATCAACCCAGG
rta3-Down	CTAATGTCTATTCCCCTGGCCAG
rta3-P1	ACCTTGACCCTTCTCTTCTTG
rta3-P2	TGGCCGGTGGATGATGTTGATTTGA
rta3-P3	GGTGAAGAGCATTGTTTGAGGCGTCTGGTATGACCAGGGGTTG
rta3-P4	CATCAGGCCTCCTCTCAGACAGCATCGAATGTATACCATTCTTGTCC
rta3-P5	AACCATCCTGCTGTCCGTGATCTCT
rta3-P6	CTTACCGATTCTTCACTCCAACTCC
rta3-Up	ATGGCCAGCTACAGTACCTGC
rta3-Down	CTAGAGCTGGTTCTTGGAACCC
rta4-P1	CCGCTCCAATACACAGTCAGG
rta4-P2	GATTGCCAGAGCATGCGTCTG
rta4-P3	GGTGAAGAGCATTGTTTGAGGCCATTGCCGGAATACTGTTTCAGC
rta4-P4	CATCAGGCCTCCTCTCAGACAGGATCAAATCCTATCGGTTGAGGG
rta4-P5	GGGCAAGGAGGGTGCAACTTACAG
rta4-P6	CAGTTGGCAGGGATGGAGT
rta4-Up	GCAAAGTTAGAGCCATACAGAGGC
rta4-Down	GCCCATCCTCATGTCCAATGCC
rta5-P1	GAAGAAGATTTGGACGAAGAGG
rta5-P2	GAAGAGAAGGAAGCAGAAGGTTCC
rta5-P3	GGTGAAGAGCATTGTTTGAGGCCATTGTGGAATGAACGTCGAG
rta5-P4	CATCAGGCCTCCTCTCAGACAGGCAGCCTAAATATTGATGACC
rta5-P5	GCTGTGCAGGATGCACATCTTG
ra5-P6	GGAATGACTGCTGGAAGTAAATC
rta5-Up	GACGACAGGAGCAGATGCCAGCTAC
rta5-Down	GGAGGAATCCTGCTCGAGGCTTTTC
rta6-P1	GAAGAAGATTTGGACGAAGAGG
rta6-P2	GAAGAGAAGGAAGCAGAAGGTTCC
rta6-P3	GGTGAAGAGCATTGTTTGAGGCCATTGTGGAATGAACGTCGAG
rta6-P4	CATCAGGCCTCCTCTCAGACAGGCAGCCTAAATATTGATGACC
rta6-P5	GCTGTGCAGGATGCACATCTTG
rta6-P6	GTGGAATGGAACGAAGATAGAAAG
rta6-Up	GATACAGGGGATTCTGAGGAGGC
rta6-Down	CCTCACCAGGGAGCTTGCATATGG
rta7-P1	CCTGTTTTAGAGTGGTCGTGGG
rta7-P2	CTACTTCTTGGCCATACCTCAAAG
rta7-P3	GGTGAAGAGCATTGTTTGAGGCCATAATCTTCGGTTGTTTCTGTCC
rta7-P4	CATCAGGCCTCCTCTCAGACAGCGCCATACAGATGATTCTCATTGG
rta7-P5	GCTGCGTATCTTCACAATGTGG
rta7-P6	CGTTCGTATCCTGTCGTCCG
rta7-Up	CCCCATATCACAATTGATGTTCC
rta7-Down	GCGGCAACGTCTCATCTCCTAG
rta5^C^-F	ACCTGCAGGCATGCAAGCTTGGCAACACAAAGAAAGCAGGA
rta5^C^-R	CGACGGCCAGTGCCAAGCTTCTAACTCATTAATGATAGGTC
rta7^C^-F	ACCTGCAGGCATGCAAGCTTCTGTATCTTGGCCCTGTCTCT
rta7^C^-R	CGACGGCCAGTGCCAAGCTTTTGTTCCTGTCTCGATGATGTTCA
M13F	GTAAAACGACGGCCAGT
M13R	CAGGAAACAGCTATGAC
pyrG-F	GCTCGAGCATGCATCTAGAGG
*pyrG-R* OE::*oxrA*-F	CTGTCTGAGAGGAGGCACTGATG CCTTTAATCAAGCTTATCGATATGTCTTCCAAGATAGTCATC
OE::*oxrA*-R	CTCGAGGTCGACGGTATCGATCTATCCGACCACGCGAACCAAATG
RT-tubA F	TTCCGTCCCGACAACTTCGT
RT-tubA R	TCACAGCCTTCAGCCTCACG
RT-*oxrA* F RT-*oxrA* R	GTCGTCAAGACCATTCACA ACAGCGGAGTTCAGAGAA
RT-012160 F	CTACCCGTTCGGCAGAGGAT
RT-012160 R	GGGCGATGTGGATAAGTAAAAT
RT-047000 F	CGTCAGCAGGGGCCTTTTT
RT-047000 R	CCGATGGCACGGAACAA
RT-050790 F	ATCATCAGCCCCAGCACAT
RT-050790 R	TCATAGCAAACATCACGCCA
RT-abcA F RT-abcA R	CGGGCTTTTGGATTTTCATGTACC TCAATATCTGAGCACTTGACGCTGG
RT-abcD F	CAGAAGCAACGCATCGCCAT
RT-abcD R	CTCTTGGACAATAGCCTCCGACT
RT-atrA F	GCATCCACGAGTCCAAGCGA
RT-atrA R	CCGCGCATATGCCAAGCATC
RT-atrB F	CTGGCCTCGACGGTCAATCC
RT-atrB R	TTGGCCAACAGCAACAGGGT
RT-abcC F	CGAGTATGCCGCCAAATCCG
RT-abcC R	CGCATCTGCCGAACATCCG
RT-fmpD F	CAGAA GCAACGCATCGCCAT
RT-fmpD R	CTCTTGGAC AATAGCCTCCGACT
RT-abcE F	GCCACCGATCCAAAGCAGGT
RT-abcE R	TGTGCATGGTAAGGCGGCAA

### Overexpression of the *oxrA* gene

Overexpression of the *oxrA* gene was achieved by inserting the full-length open reading frame (ORF) of *oxrA* into the pBARGPE plasmid. This plasmid harbors a strong *gpdA* promoter (to drive high-level transgene expression) and multiple unique, commercially available restriction enzyme sites for flexible cloning.Genomic DNA was isolated from the wild-type (WT) *Aspergillus fumigatus* strain A1160 and used as a template for PCR amplification. A DNA fragment containing the complete *oxrA* ORF was amplified using the gene-specific primer pair OE-oxrA-F/OE-oxrA-R; a *Cla*Ⅰ restriction site was pre-introduced at the 5’ end of both primers to facilitate subsequent cloning.Subsequently, the pBARGPE plasmid was digested with *Cla*Ⅰ restriction endonuclease (under manufacturer-recommended reaction conditions) to generate linearized vector. The digested plasmid was purified using a commercial DNA purification kit to remove residual enzyme and genomic contaminants, yielding a clean linearized vector. This linearized vector—carrying the *pyrG* selectable marker—was then recombined and ligated with the purified *oxrA* ORF fragment using DNA ligase.The resulting recombinant ligation product was transformed into competent *Escherichia coli* cells via heat shock. Positive recombinant clones were initially screened by colony PCR using the primer pair GPD-F/OE-oxrA-R.Clones verified as positive were subjected to large-scale liquid culture in LB medium supplemented with appropriate antibiotics. Plasmids were extracted from these cultures using a high-purity plasmid extraction kit. Finally, the purified *oxr* overexpression plasmid was introduced into the recipient *A. fumigatus* strain A1160 via a standard protoplast transformation protocol [54].

### RNA sequencing analysis

For RNA-seq analysis, 1 × 10^8^ conidia of each relevant strain were inoculated into liquid Minimal Medium (MM). The cultures were incubated in a rotary shaker at 37 °C with 220 rpm shaking for 18 h. Mycelia were then collected and flash-frozen in liquid nitrogen.Three biological replicates were set up for each sample of the wild-type (WT) strain, *Δrta5* strain, and *Δrta7* strain, as well as the stimulation group and non-stimulation group of wild-type strains treated with Itraconazole(ITC). These samples were sent to Paisano Biotechnology Co., Ltd. (Nanjing, China) for transcriptome analysis using the Illumina platform. The raw data have been deposited in the Sequence Read Archive (SRA) of the National Center for Biotechnology Information (NCBI; URL: https://www.ncbi.nlm.nih.gov/sra) with accession numbers PRJNA1369192 and PRJNA1367209. Specifically, PRJNA1369192 includes the sequencing data of three biological replicates for WT, *Δrta5* and *Δrta7* strains, and PRJNA1367209 includes the sequencing data of three biological replicates for WT and ITC-treated strains.

### RNA isolation and quantitative real-time PCR (qRT-PCR) assay

Sample preparation for the qRT-PCR assay followed the same procedure as described above for RNA-seq. The primers used for qRT-PCR in this study are detailed in Table 2. Total RNA was extracted using the UNIQ-10 Column Total RNA Extraction Kit (Sangon Biotech Co., Ltd., Shanghai, China). Subsequent digestion and reverse transcription were conducted employing the HiScriptII Q RT SuperMix for qPCR (gDNA wiper) kit to synthesize complementary DNA (cDNA) (Vazyme Biotech Co., Ltd.). Quantitative PCR (qPCR) experiments were performed using AceQ qPCR SYBR Green Master Mix (Vazyme Biotech Co., Ltd.) on a LightCycler 480 instrument (Roche). The expression level of the *tubA* gene was used as an internal reference for normalization. The relative gene expression levels were calculated using the 2⁻^ΔΔCT^ method.

### Statistical analysis

All data obtained in this study were used for graph generation, data distribution analysis, and statistical analysis using GraphPad Prism 10.0. An unpaired Student’s t-test was used for comparisons between two groups, and one-way analysis of variance was employed for comparisons among multiple groups. For assumption checks, the Shapiro–Wilk test was used to test normality of each dataset and the Brown-Forsythe test was used to assess the homogeneity of variances. The significance were calculated using one-way ANOVA with Fisher’s Least Significant Difference of post hoc test. All experiments were performed in three independent replicates (*n* = 3). A *P*-value of < 0.05 was considered statistically significant. The significance levels were denoted as follows: “ns” for not significant; * for *P <* 0.05; ** for *P* < 0.01; ***for *P* < 0.001; and ****for *P* < 0.0001.

## Supplementary Information

Supplementary Material 1. Fig. S1. Colony characteristics of Rta deletion mutant strains. **A** Colony phenotypes of the Rta deletion mutant strains and the WT strain on solid minimal medium (MM) and yeast extract agar glucose medium (YAG) at 37°C for 2 days ,and in liquid MM at 37°C with shaking (220 rpm) for 1 day; **B** The colony diameter statistical analysis of the indicated strains; **C** Quantitative analysis of conidial production on solid MM and (D) on solid YAG; **E** The quantitative analysis of the biomass for the Rta deletion mutant strains.

Supplementary Material 2. Fig. S2. Deletion of relevant rta genes does not affect the osmotic stress resistance of *Aspergillus fumigatus. *Colony morphology and corresponding quantitative analysis of WT and Δrta deletion strains (Δrta1, Δrta3, Δrta4, Δrta5, Δrta6, Δrta7)) grown on MM plates supplemented with 1 M KCl, 1 M NaCl, or 1.2 M sorbitol at 37 °C for 2 days. Data are presented as the mean ± standard deviation (SD) of three independent biological replicates. Statistical significance was determined using an unpaired two-tailed Student’s t-test. (ns, not significant).

Supplementary Material 3. Fig. S3. Deletion of rta1 increases the transcript level of *oxrA* in *Aspergillus fumigatus*. Relative mRNA transcript level of *oxrA* in the WT and Δrta1 strains, as determined by qRT-PCR. Data are presented as the mean ± standard deviation (SD) of three independent biological replicates. Statistical significance was determined using an unpaired two-tailed Student’s t-test.(***,*P* < 0.001).

Supplementary Material 4. Fig. S4. Expression level of the azole drug target Cyp51 in rta gene deletion strains. Differential expression gene analysis of transcriptomic data revealed that Cyp51 expression in Δrta5 and Δrta7 strains under azole treatment.(ns, not significant).

## Data Availability

The RNA-seq datasets generated and analysed during the current study are available in the NCBI Sequence Read Archive (SRA) repository, accession number: PRJNA1367209 and PRJNA1369192.
