# Supplementary figures and images for: RTA-like proteins regulate azole susceptibility by affecting the expression of the oxidoreductase gene oxrA in Aspergillus fumigatus
Source: BMC Microbiol. 2026 Apr 13;26:489. doi: 10.1186/s12866-026-04985-x (PMC13200435; doi:10.1186/s12866-026-04985-x)

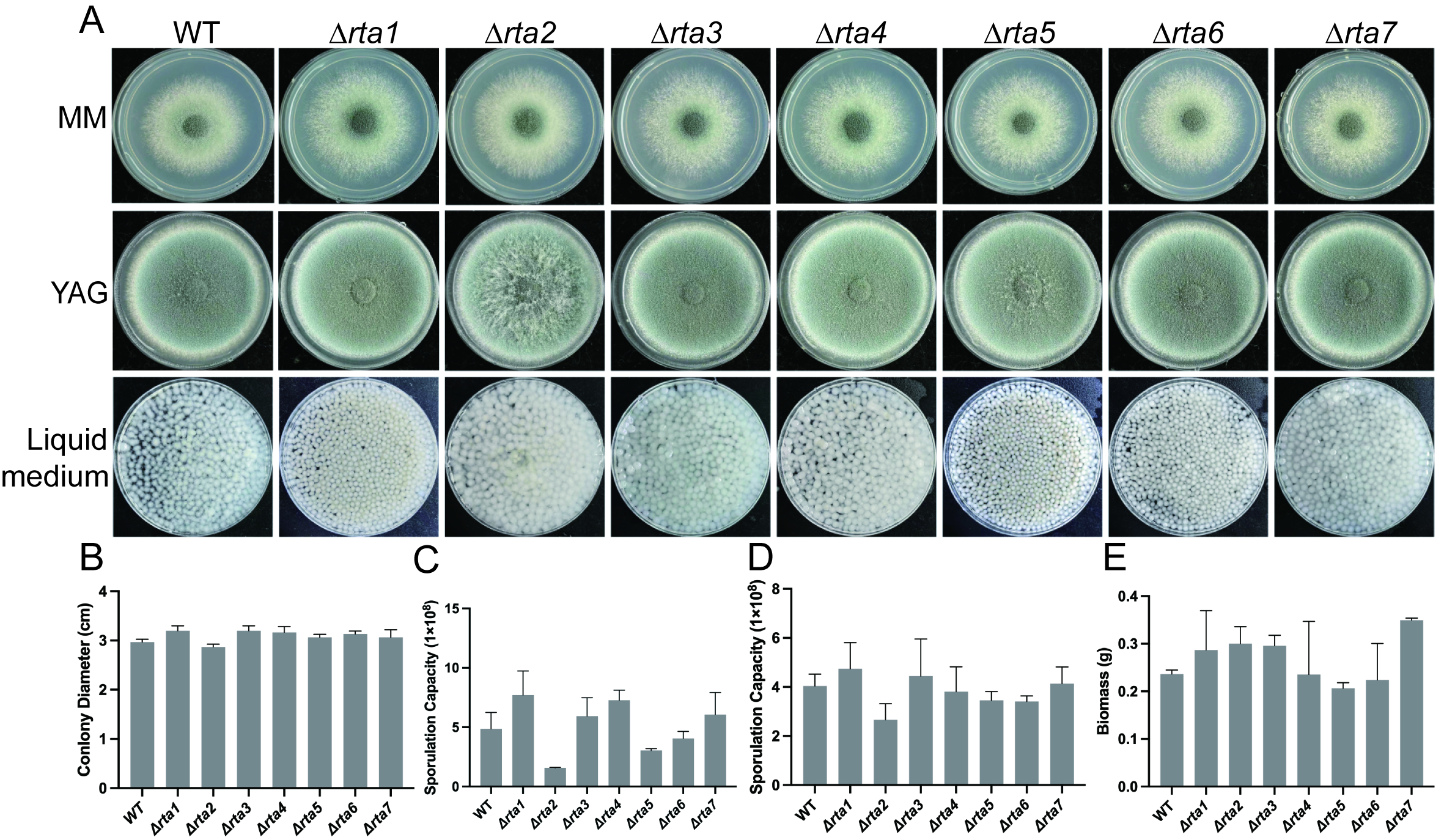

Supplement: Supplementary file 1 — Supplementary Material 1. Fig. S1. Colony characteristics of Rta deletion mutant strains. A Colony phenotypes of the Rta deletion mutant strains and the WT strain on solid minimal medium (MM) and yeast extract agar glucose medium (YAG) at 37°C for 2 days ,and in liquid MM at 37°C with shaking (220 rpm) for 1 day; B The colony diameter statistical analysis of the indicated strains; C Quantitative analysis of conidial production on solid MM and (D) on solid YAG; E The quantitative analysis of the biomass for the Rta deletion mutant strains. [file 12866_2026_4985_MOESM1_ESM.tif]

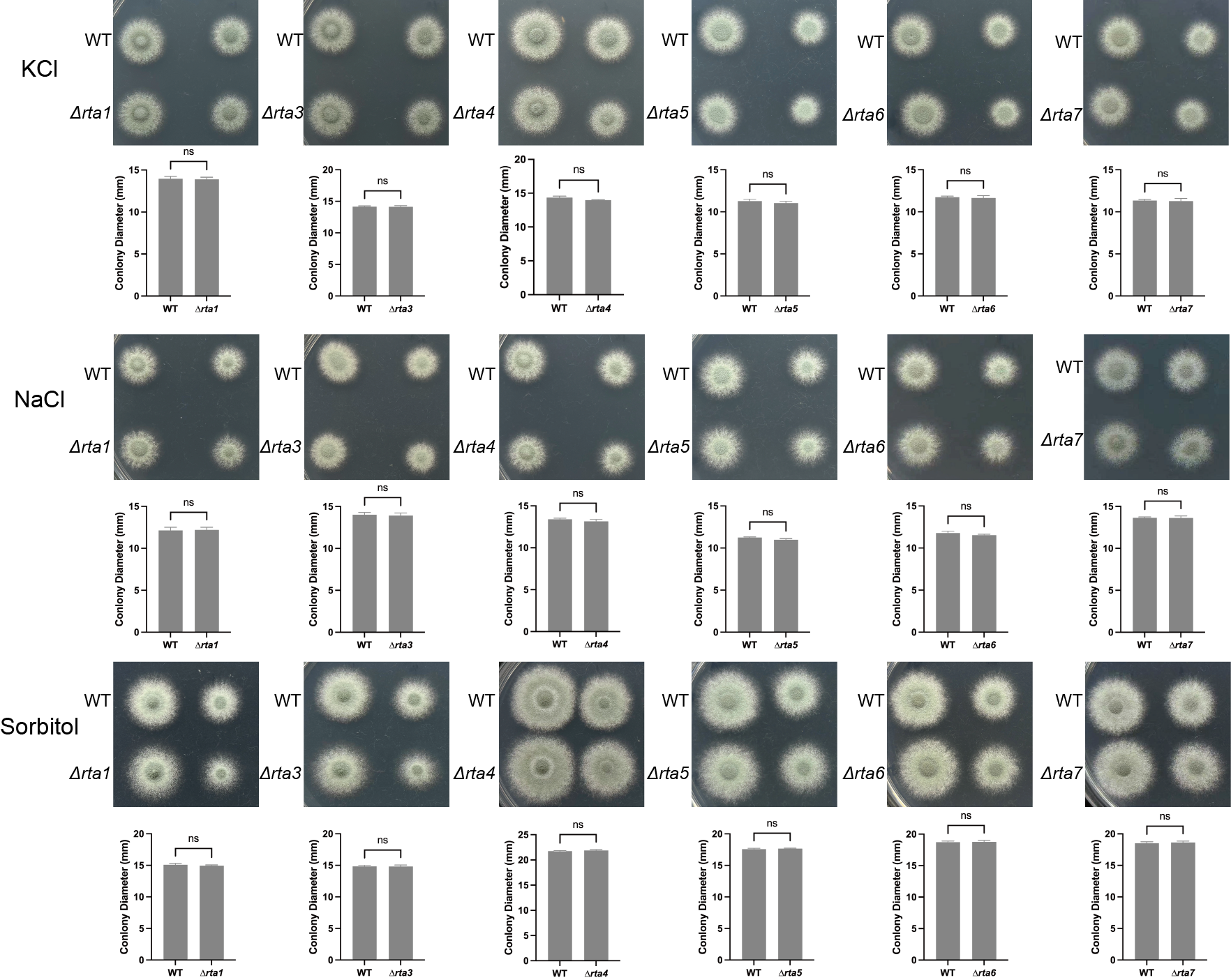

Supplement: Supplementary file 2 — Supplementary Material 2. Fig. S2. Deletion of relevant rta genes does not affect the osmotic stress resistance of Aspergillus fumigatus. Colony morphology and corresponding quantitative analysis of WT and Δrta deletion strains (Δrta1, Δrta3, Δrta4, Δrta5, Δrta6, Δrta7)) grown on MM plates supplemented with 1 M KCl, 1 M NaCl, or 1.2 M sorbitol at 37 °C for 2 days. Data are presented as the mean ± standard deviation (SD) of three independent biological replicates. Statistical significance was determined using an unpaired two-tailed Student’s t-test. (ns, not significant). [file 12866_2026_4985_MOESM2_ESM.png]

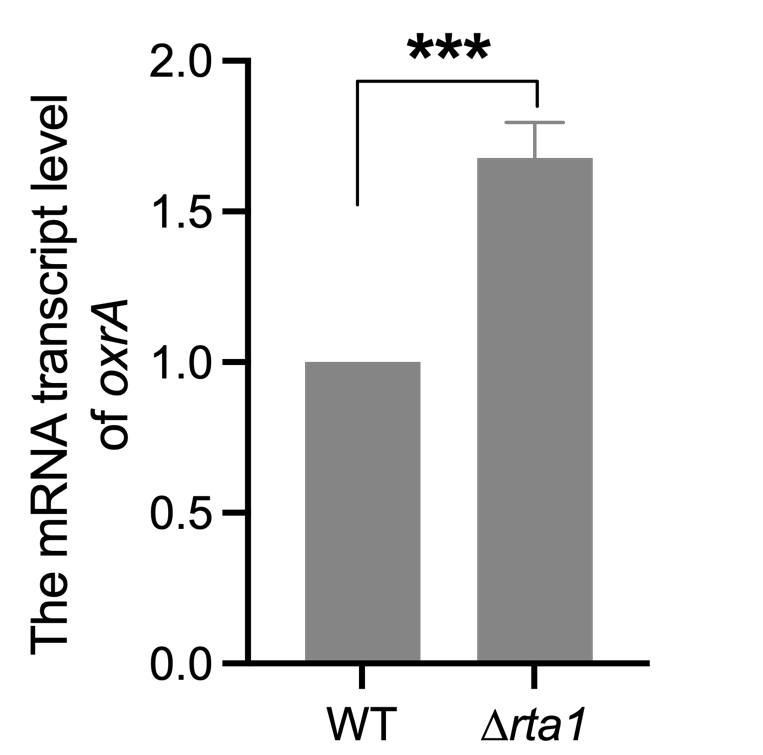

Supplement: Supplementary file 3 — Supplementary Material 3. Fig. S3. Deletion of rta1 increases the transcript level of oxrA in Aspergillus fumigatus. Relative mRNA transcript level of oxrA in the WT and Δrta1 strains, as determined by qRT-PCR. Data are presented as the mean ± standard deviation (SD) of three independent biological replicates. Statistical significance was determined using an unpaired two-tailed Student’s t-test.(***,P < 0.001). [file 12866_2026_4985_MOESM3_ESM.tiff]

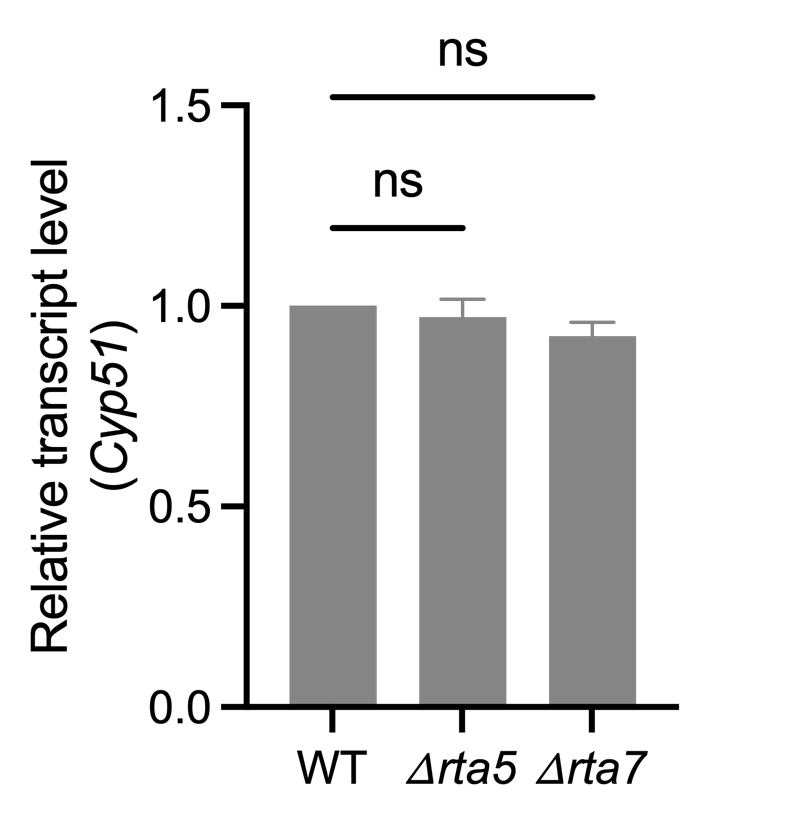

Supplement: Supplementary file 4 — Supplementary Material 4. Fig. S4. Expression level of the azole drug target Cyp51 in rta gene deletion strains. Differential expression gene analysis of transcriptomic data revealed that Cyp51 expression in Δrta5 and Δrta7 strains under azole treatment.(ns, not significant). [file 12866_2026_4985_MOESM4_ESM.tiff]
